# Near-Infrared Spectroscopy in Schizophrenia: A Possible Biomarker for Predicting Clinical Outcome and Treatment Response

**DOI:** 10.3389/fpsyt.2013.00145

**Published:** 2013-11-14

**Authors:** Shinsuke Koike, Yukika Nishimura, Ryu Takizawa, Noriaki Yahata, Kiyoto Kasai

**Affiliations:** ^1^Office for Mental Health Support, Division for Counseling and Support, The University of Tokyo, Tokyo, Japan; ^2^Department of Neuropsychiatry, Graduate School of Medicine, The University of Tokyo, Tokyo, Japan; ^3^Social, Genetic and Developmental Psychiatry Centre, Institute of Psychiatry, King’s College London, London, UK; ^4^Department of Youth Mental Health, Graduate School of Medicine, The University of Tokyo, Tokyo, Japan

**Keywords:** near-infrared spectroscopy, verbal fluency task, biological markers, early intervention, clinical outcome

## Abstract

Functional near-infrared spectroscopy (fNIRS) is a relatively new technique that can measure hemoglobin changes in brain tissues, and its use in psychiatry has been progressing rapidly. Although it has several disadvantages (e.g., relatively low spatial resolution and the possibility of shallow coverage in the depth of brain regions) compared with other functional neuroimaging techniques (e.g., functional magnetic resonance imaging and positron emission tomography), fNIRS may be a candidate instrument for clinical use in psychiatry, as it can measure brain activity in naturalistic position easily and non-invasively. fNIRS instruments are also small and work silently, and can be moved almost everywhere including schools and care units. Previous fNIRS studies have shown that patients with schizophrenia have impaired activity and characteristic waveform patterns in the prefrontal cortex during the letter version of the verbal fluency task, and part of these results have been approved as one of the Advanced Medical Technologies as an aid for the differential diagnosis of depressive symptoms by the Ministry of Health, Labor and Welfare of Japan in 2009, which was the first such approval in the field of psychiatry. Moreover, previous studies suggest that the activity in the frontopolar prefrontal cortex is associated with their functions in chronic schizophrenia and is its next candidate biomarker. Future studies aimed at exploring fNIRS differences in various clinical stages, longitudinal changes, drug effects, and variations during different task paradigms will be needed to develop more accurate biomarkers that can be used to aid differential diagnosis, the comprehension of the present condition, the prediction of outcome, and the decision regarding treatment options in schizophrenia. Future fNIRS researches will require standardized measurement procedures, probe settings, analytical methods and tools, manuscript description, and database systems in an fNIRS community.

## Introduction

Techniques that allow the easier and less invasive measurement of brain structure and activity, such as magnetic resonance imaging (MRI), functional MRI, and positron emission tomography (PET), have progressed rapidly over the past 20 years. There has been considerable expectation regarding the clinical application of neuroimaging techniques to psychological conditions and psychiatric illnesses (1, 2). Biological markers measured using neuroimaging instruments would clarify the pathophysiological features of psychiatric disorders, inform patients and family members regarding their actual conditions, and improve general impression of psychiatric disorders, by more easily giving the explanation of their conditions and discussing their assumed prognoses.

**Figure 1:**
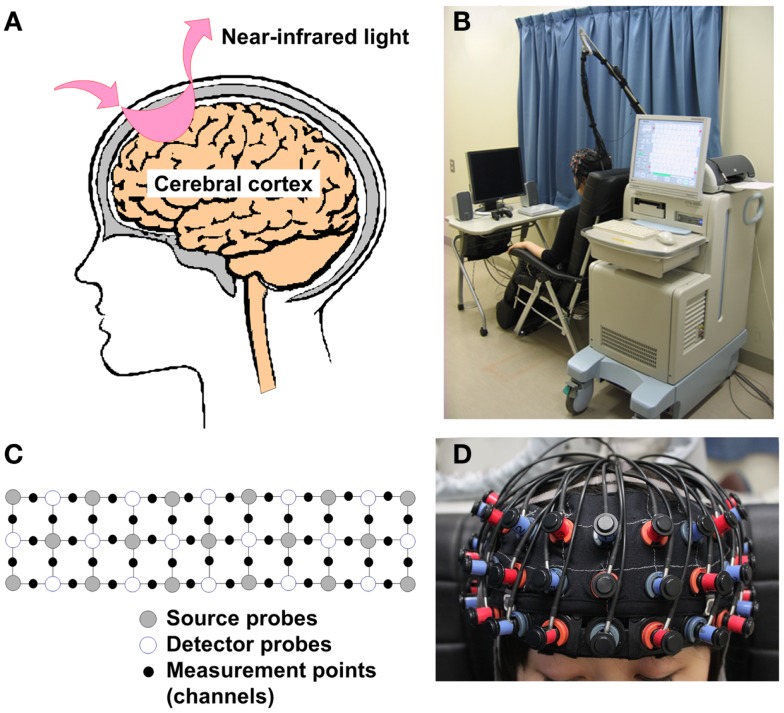
**Theoretical background and measurement settings of fNIRS**. **(A)** Illustration of a simple source and detector probe set. When near-infrared light is emitted from a source probe onto the human scalp, it passes and scatters through brain tissues with relatively low absorption. Subsequently, part of this light is absorbed by blood hemoglobin in small vessels. A detector probe, normally placed 3 cm away from the source probe in adults, can detect scattered near-infrared light reflected by the surface of the cortex. **(B)** fNIRS measurement setting (ETG-4000, Hitachi Medical Corporation). Commercial fNIRS machines are small, movable, and work silently, so that they are easy to use in clinical settings. **(C,D)** 3 × 11 Probe attachment and fitting condition. Multi-channel fNIRS machines use multiple sources and detector probes and are able to measure brain activity between the probes. **(A–C)** Was approved by Hitachi Medical Corporation.

Functional near-infrared spectroscopy (fNIRS) is a functional brain imaging tool that can measure hemoglobin changes over the surface of the brain easily and non-invasively (Figure 1) (3–5). fNIRS technique was found in 1977 (6), and has been applied to measure brain hemodynamic activity through the scalp (7, 8). The release of commercial fNIRS machines that are small, movable, and work silently during the last decade (Figure 1B) has allowed the progress of fNIRS research in the field of psychiatry (9). In 2012, more than 100 studies were published on this subject; among these, about 20 articles pertained to the field of psychiatry (9). Part of the results of those fNIRS studies has been approved as one of the Advanced Medical Technologies as an aid for the differential diagnosis of depressive symptoms by the Ministry of Health, Labor and Welfare of Japan in 2009 (10–12), as the presence of different characteristic waveform patterns in the prefrontal cortex (PFC) during a verbal fluency task (VFT) has been reported among patients with major depressive illness (4, 12–17), bipolar disorder (12, 13), and schizophrenia (4, 5, 12). This was the first such approval in the field of psychiatry in Japan (see the “Application to supplementary diagnostic tool for psychiatric disorders” subsection). Here, we reviewed fNIRS research that focused on schizophrenia, which is currently the most published topic in the application of fNIRS in the field of psychiatry, and address future investigations that are needed for the clinical application of this technique as an aid for the differential diagnosis, comprehension of present conditions, prediction of outcome, and decision regarding treatment options in schizophrenia.

## Principles of Brain Activity Measurement Using Near-Infrared Light

### Theoretical background of the measurement of brain activity using fNIRS instruments

Near-infrared light, especially that with a wavelength of 650–1000 nm, has characteristics that include a relatively high absorption through hemoglobin, as well as a relatively high penetration through bone and skin, compared with light with other wavelengths. The emission of near-infrared light from a source probe on the human scalp leads to its passing and scattering through brain tissues with relatively low absorption, followed by the absorption of part of this light by blood hemoglobin in small vessels (<1 mm) (18). A detector probe, which is normally placed 3 cm away from the source probe in adults, can detect scattered near-infrared light that is reflected by the surface of the cortex (Figure 1A). Therefore, the region located between the source and the detector probes is generally set as a measurement area that is often called a “channel” (Figure 1C). fNIRS instruments can measure oxygenated hemoglobin (O_2_Hb) and deoxygenated hemoglobin (HHb), as well as total hemoglobin (tHb) by summing up O_2_Hb and HHb, by using near-infrared light with two or more different wavelengths that are slightly different from the absorption rates of O_2_Hb and HHb. In accordance with light manipulation, fNIRS instruments are roughly divided into three types: continuous wave (CW), frequency domain (FD), and time domain (TD) instruments. Although CW-type fNIRS machines are unable to measure absolute hemoglobin concentration in tissues, they are relatively small, have low installation and maintenance costs, and are able to perform measurements at a high sampling rate compared with FD- and TD-type fNIRS machines (18). Therefore, recent clinical studies have used CW-type fNIRS machines (19, 20). The theoretical details of all types of fNIRS instruments were reviewed elsewhere (18, 20, 21).

### Advantages and disadvantages of fNIRS compared with other functional imaging instruments

The advantages and disadvantages of CW-type fNIRS compared with other functional imaging instruments (fMRI, PET, and EEG) are shown in Table 1. fNIRS has the following advantages: (1) non-invasiveness, which allows repetitive measurements, even in infants (21, 22); (2) easy setting; (3) small size and portability; (4) high temporal resolution compared with fMRI and PET (18, 20); (5) possibility of performing measurements in a non-restrained position, such that participants can sit on a chair, talk, and move their hands; and (6) possibility of relatively easily combining measurements with other neuroimaging techniques, such as EEG (23, 24), MRI (25–30), PET (31–33), and magnetoencephalography (MEG) (34, 35). Conversely, fNIRS has the following disadvantages: (1) low spatial resolution (10–30 mm); (2) possibility of performing measurements only at the surface of the cortex; (3) inability to measure absolute hemoglobin value (CW-type); and (4) the data obtained can be influenced by scalp, muscle, skull, and cerebrospinal fluid factors in addition to hemodynamic changes in the cortex (20).

**Table 1:** **Comparison of CW-type fNIRS machines with other neuroimaging tools**.

		fNIRS	fMRI	PET	EEG	MEG
Theoretical background	Signal property	Scattered near-infrared light	Magnetic property	Uptake of ligand marked positron	Collection of neural activity	Magnetic fields produced by brain’s electrical activity
	Measurement area	Surface of the cortex	**Whole brain region**	**Whole brain region**	Surface of the cortex unless using depth EEG	Surface of the cortex
	Time resolution (s)	1	2–3	≥10	**0.01**	**0.01**
	Spatial resolution (mm)	20	**5**	10	20	10
	Effect of extra-cortical tissue	Some	**Little**	**Little**	Some	None
Measurement setting	Invasiveness	**No**	**No**	Intravenous injection of radioactive ligand	**No**	**No**
	Body movement	**Tolerable**	No	No	No	No
	Head restraint	**No**	Yes	Yes	**No**	Yes
Instrument	Size	**Small, movable**	Large, fix	Large, fix	Large in research use	Large, fix
	Transportability	**Yes**	No	No	Limited	No
	Initial cost	**300,000–400,000 USD**^a^	Several million USD	Several million USD	**100,000–300,000 USD**	Several million USD
	Measurement and maintenance cost	**Reasonable**	Moderate	Very expensive (positron ligand)	**Reasonable**	Moderate

*Bold shows advantages compared to other neuroimaging tools*.

*^a^ ETG-4000 (Hitachi Medical Corporation)*.

Other than task-related hemoglobin changes derived from neural activities under neurovascular coupling, fNIRS signals vary depending on task-related blood pressure changes and skin blood flow, as well as spontaneous brain activity related to heart rate, respiration, and physiological oscillations (20, 21). Simultaneous measurements by fNIRS and pulse Doppler sonography or by fNIRS with different probe distances [e.g., shallow (5 mm) and deep (30 mm)] allow the distinction of fNIRS signals from the cortex from those of other brain tissues. A study based on fNIRS with different probe distances and laser Doppler velocimetry showed that hemoglobin changes from the cortex during the VFT may contain only 6% of fNIRS data in the PFC, and that most signal changes may arise from changes in skin blood flow (36). The results OPF simultaneous fNIRS, fMRI, MR angiography, and peripheral physiological measurements suggested that task-evoked sympathetic arterial vasoconstriction affected fNIRS signal changes substantially (37). However, a subsequent study in which simultaneous measurements using multiple fNIRS probe distances and laser Doppler flowmetry were performed suggested that about 50% of fNIRS signals contributed to the fNIRS signal component in the deep layer, mostly measured in the cortex, during several cognitive tasks, including the VFT (38). A study of simultaneous measurement using fMRI, fNIRS, and lase Doppler flowmeter showed that the prefrontal fNIRS signals were significantly correlated with the blood oxygenation level-dependent (BOLD) signals in the gray matter rather than those in the soft tissue or the laser Doppler signals (39). Previous studies have suggested that the ratio of fNIRS signal changes in the cortex varies from 6 to 60%, and the variation has been considered as being caused by differences in measuring instruments, estimation methods, and brain areas measured (20, 36–38, 40–44). Several studies have provided filtering methods that allow raising the ratio of task-related hemoglobin changes in the cortex to in the extra-cortical tissues; however, these systems require additional probes and complex analytical methodologies because of different near-infrared absorption and scattering coefficients in each tissue and anatomic characteristics throughout the light path in each brain area (25, 38, 40, 41). Therefore, fNIRS is a reliable tool for research based on the group-level and/or channel-cluster-level investigations, although its reliability at the individual and single-channel levels is not sufficient (18, 45–48). Few studies in clinical psychiatry have considered these filtering methods because of the limitations of measurement time and setting. Future studies will be needed to improve the signal-to-noise ratio for application over wide measurement areas without losing the advantage of fNIRS instruments.

### Comparison of fNIRS hemoglobin changes with BOLD signals in fMRI

The measurement of brain activity using the fMRI technique has progressed during the last two decades. The BOLD signal is thought to represent the differences in the magnetic properties of deoxygenated hemoglobin concentration under T2-weighted measurement conditions when cerebral blood flow (CBF) increases and HHb decreases in small vessels (49). The theoretical model of BOLD compared with fNIRS signals was reviewed in detail elsewhere (18). Briefly, localized O_2_Hb decreases and HHb increases occur (i.e., initial dip) when neurons are activated in a specific region. Several seconds later, the blood flow system is triggered to request glucose to the region (i.e., hemodynamic response), which is followed by a CBF increase and peripheral vascular bed dilation, leading to tHb increase and HHb decrease in small vessels, and O_2_Hb increase in blood capillaries and vascular bed. As this neurovascular coupling that occurs in the activated area is thought to vary according to brain area and vessel diameter, and to be sensitive to persistent neural activity, the relationship between hemoglobin changes and BOLD/fNIRS signals has a complex variation pattern.

Other than the difference in spatio-temporal measurement characteristics between BOLD and fNIRS signals, the BOLD signal is thought to detect mainly changes in the magnetic properties of small vessels, whereas the fNIRS signal is thought to detect changes in near-infrared light absorption in blood capillaries (50). Therefore, several discrepancies may occur between BOLD and fNIRS signals. Previous fNIRS studies have yielded inconsistent results compared with other imaging tools. Simultaneous fNIRS and fMRI studies have shown that the BOLD signal in a specific region was associated with the O_2_Hb of fNIRS signal changes in the corresponding region (25, 29, 39, 51) or with HHb changes (26–28, 30, 51). One of the explanations for these inconsistent correlation results is that most fNIRS studies have been conducted using block-designed tasks, whereas fMRI studies have mainly used an event-related design. In addition, the analysis of fMRI data by software such as Statistical Parametric Mapping (SPM) uses a hypothesis that fits a probable activation model, whereas most fNIRS studies have analyzed average signal intensity during the whole task period without any probable activation model. Differences in acquired signal handling may result in discrepancies regarding regions with significant brain activity. As O_2_Hb data generally exhibit larger changes compared with HHb during cognitive activity, most clinical fNIRS studies have mainly been analyzed using O_2_Hb data.

### Estimation of brain area

As more studies have focused on the spatial characteristics of brain activity using multi-channel fNIRS instruments, the need to clarify the estimated location of each probe in the cortex and each channel on the scalp has arisen. As reviewed by Tsuzuki et al. recently (52), several methodologies can estimate brain areas at each probe and channel, such as structural MRI measurement using fNIRS probe marks for each participant (53, 54), a probabilistic registration method using a 3D digitizer (55), and a probabilistic virtual registration method without any additional instrument (56). As the virtual registration method enables the estimation of brain areas based on standard brain images at each probe and channel by defining only probe setting based on the 10–20 system electrode locations, and because this method has similar accuracy compared with other estimation methodologies (56), most fNIRS studies have applied this method to estimate the measurement brain areas of channels. fNIRS software can also use the virtual registration method as a toolbox (http://www.jichi.ac.jp/brainlab/tools.html) (57). There are several standard stereotaxic coordinate systems such as the Montreal Neurological Institute (MNI) and the Talairach Daemon. The LONI Probabilistic Brain Atlas (LPBA40) (58) system has been often used in fNIRS studies based on probabilistic registration methods (3, 52, 59, 60).

## Application of fNIRS in Schizophrenia Research

Schizophrenia is a syndrome that is characterized by positive and negative symptoms and cognitive dysfunction with enduring social deficits. Moreover, it affects approximately 0.7% of the general population (2). The World Health Organization reported that the estimated burden of schizophrenia accounts for 2.3% of all diseases worldwide, and its disability-adjusted life year ranks ninth among all non-communicable diseases (61). However, effective treatments and objective indices for all symptoms and functions of schizophrenia have not been fully met.

Since the first fNIRS report of altered activation patterns in schizophrenia compared with healthy controls was published in 1994 (62), fNIRS research focusing on schizophrenia has been the most published topic in the field of psychiatry (9). We reviewed systematically research articles published up to April 1, 2013, by searching PubMed and Web of Science. As in previous systematic reviews (9, 18), we used “[(near infrared) OR (optical topography)] AND (schizo* OR psycho*) AND (brain OR cortex)” as a search term. Two hundred and sixty articles were extracted, among which 29 explored brain activity in patients with schizophrenia. Half of these articles (15 articles, including 4 studies of genetic variants) adopted a VFT as an activation cognitive battery during measurement.

### Verbal fluency task

The VFT is a popular cognitive task that is used in neuropsychological tests and neuroimaging measurements to explore various cognitive functions during verbal recall, retrieval, working memory, attention, and inhibition (avoiding inappropriate words) (63, 64). During the task, participants are instructed to say as many words from a given paradigm as possible in a given time (usually 60 s). This paradigm is roughly divided into semantic (category fluency task, CFT), such as fruits, or phonological (letter fluency task, LFT), such as words that begin with the letter “p.” Neuropsychological studies have revealed that patients with schizophrenia have worse VFT performances compared with healthy controls (65). Although fMRI and fNIRS studies have shown that relatively global brain activity occurs during the VFT compared with a task that requires specific cognitive domains, such as the n-back working-memory task (28, 66) and the go/no-go task (67), most participants (including patients with chronic schizophrenia) can perform the task (10, 11, 19, 63, 64).

Eleven previous VFT studies are listed in Table 2. Watanabe and Kato firstly described that patients with schizophrenia had reduced O_2_Hb and HHb changes in the left PFC during the LFT compared with healthy controls (68). This study also demonstrated that patients who were medicated with atypical antipsychotics had better task performances and similar O_2_Hb changes compared with controls, suggesting that typical antipsychotics may impair task response and brain activity. However, that study did not explore whether impaired O_2_Hb changes were derived from impaired task response or functional impairment in the PFC, which may be ameliorated by atypical antipsychotics. Suto et al. firstly described the spatio-temporal activity patterns in the PFC and temporal cortex among patients with depression and schizophrenia and healthy controls using two 24-channel fNIRS machines (4). In that study, a modified task procedure was adopted in which three initial syllables changed in turn every 20 s during a 60 s task period, to help participants avoid silence and reduce differences in task performances among groups. Patients with schizophrenia had lower activity in the bilateral PFC and temporal cortex at the start of the task period compared with controls, whereas patients with depression had lower activity in the bilateral PFC and temporal cortex across the task period. These results were irrespective of task performance, and the task paradigm used in that study was used widely in further studies. In addition, this result was based on the Advanced Medical Technologies in Japan (10–12). Using a larger cohort, Takizawa et al. replicated the observation that patients with schizophrenia had slower and inappropriate activity after the task period compared with healthy controls (5). Furthermore, activities in the frontopolar prefrontal cortex (FPC) region were positively associated with global assessment of functioning scores in schizophrenia. Quaresuma et al. replicated the finding of reduced brain activity in schizophrenia during the LFT, whereas no significant change was observed during a visual spatial working memory task (69). Ikezawa et al. and Azechi et al. showed the efficacy of the LFT task in fNIRS (70, 71). Ikezawa et al. measured hemoglobin changes in the PFC using a two-channel fNIRS instrument during the LFT, CFT, Tower of Hanoi (TOH), the Sternberg task, and the Stroop task, and showed that brain activities during the LFT and TOH were significantly different between patients with schizophrenia and healthy controls (71). Azechi et al. explored this further using discrimination analysis and showed that 88.3% of participants correctly discriminated between patients and controls based on task performance on the TOH, LFT, and CFT, and on fNIRS signals during the VFT (70). This result confirmed that 75% of independent participants were able to discriminate correctly using the same procedure. Koike et al. explored the signal differences among different clinical stages of schizophrenia (ultra-high risk, first-episode psychosis, and chronic schizophrenia) and showed that the activities in the FPC, ventrolateral PFC (VLPFC), and temporal cortex were lower in patients than they were in controls, whereas the activities in the dorsolateral PFC (DLPFC) decreased with advancing clinical stage (3). Those authors also replicated the finding that the activity in the FPC region was positively associated with global assessment of functioning scores in chronic schizophrenia, implying that it may be a candidate biomarker for the assessment of psychological condition in schizophrenia (3, 5). Takeshi et al. measured brain activity during the idea fluency task, which is thought to require more executive function, and showed that patients with schizophrenia had decreased activity in the ventral area of the PFC (72). Furthermore, these signal changes were positively associated with the global assessment of functioning scores, whereas O_2_Hb changes during the LFT were not. Shimodera et al. replicated the characteristic waveforms in schizophrenia, such as smaller initial activity at the start of the task period, reduced activity during the task period, and inappropriate activity after the task period, and used a numerical calculation (73).

**Table 2:** **Previous fNIRS studies in schizophrenia using verbal fluency tasks**.

Reference	Place	fNIRS instrument		Case demographics			
		Model^a^	Number of analyzed channels	Number of cases (M/F)	Mean age in cases (SD)	Medication	Recruitment places
Shimodera et al. (73)	Kochi	OMM-3000/16	42	32 (12/19)	42.4 (15.7)	All	University hospital
Koike et al. (3)	Tokyo	ETG-4000	52	38 (22/16)^b^	31.3 (6.1)^b^	All^b^	University hospital, psychiatric hospital, health service center, and clinics
Takeshi et al. (72)	Tokyo (Toho University)	OMM-3000/16	24	18 (7/11)	25.4 (5.8)	All	NA
Azechi et al. (70)	Osaka	OMM-3000/16	2	30 (16/14)^c^	39.6 (13.1)^c^	All	University hospital
Quaresima et al. (69)	L’Aquila	NIRO-300	2	9 (5/4)	32.1 (8.3)	All	NA
Ikezawa et al. (71)	Osaka	NIRO-200	2	30 (12/18)	38.7 (11.7)	Two patients drug naïve^d^	University hospital
Takizawa et al. (5)	Tokyo	ETG-4000	52	55 (26/29)	40.1 (11.1)	All	University hospital
Ehlis et al. (74)	Wuerzburg	ETG-100	22	12 (9/3)	34.2 (10.4)	One patient drug naïve	NA
Kubota et al. (75)	Cleveland	NIRO-300	2	16 (8/8)	37.5 (13.0)	All	NA
Suto et al. (4)	Gunma	ETG-100	48	13 (9/4)	37.9 (12.0)	All	University hospital
Watanabe and Kato (68)	Ehime	HEO-200	1	62 (30/32)	40.1 (12.3)	All	Psychiatric hospital

**Reference**	**Task setting**			**Results^e^**			
	**Pre-task condition**	**Task condition**	**Post-task condition**	**Patients compared to controls**		**Estimated regions for group differences**	**Multiple analysis correlation**

Shimodera et al. (73)	30-s Rest	30-s × 2 Letter sets	70-s Rest	↓		Bilateral FPC, DLPFC, and VLPFC regions	Bonferroni
Koike et al. (3)	60-s Vowel repeats	20-s × 3 Letter sets	70-s Vowel repeats	↓^f^		Bilateral FPC, DLPFC, and VLPFC regions^f^	FDR
Takeshi et al. (72)	60-s Vowel repeats	20-s × 3 Letter sets	70-s Vowel repeats	↓		Bilateral dorsal FPC and DLPFC	No
Azechi et al. (70)	30-s Vowel repeats	20-s × 3 Letter/category sets	60-s Vowel repeats	↓^g^		Bilateral FPC	No
Quaresima et al. (69)	120-s Rest	30-s × 4 Letter sets	No setting	↓		Bilateral FPC	No
Ikezawa et al. (71)	30-s Vowel repeats	20-s × 3 Letter/category sets	60-s Vowel repeats	↓^g^		Bilateral FPC	ANCOVA
Takizawa et al. (5)	60-s Vowel repeats	20-s × 3 Letter sets	70-s Vowel repeats	↓		Bilateral FPC, DLPFC, and VLPFC regions	FDR
Ehlis et al. (74)	10-s Rest	30-s Letter/category and 30-s rest × 2 sets	30-s Control task (repeatedly say weekdays) and 30-s rest between the sets of task conditions	↓^g^		Left DLPFC and VLPFC	ANOVA
Kubota et al. (75)	20-s A vowel repeats	15-s × 6 Letter/category sets	No setting	↓^g^		Bilateral FPC	ANOVA
Suto et al. (4)	30-s Vowel repeats	20-s × 3 Letter sets	60-s Vowel repeats	–		–	ANOVA
Watanabe and Kato (68)	15-s No detailed description	60-s One letter set	15-s No detailed description	↓		Left FPC	ANOVA

**Reference**	**Correlational analysis between clinical variables and fNIRS signals**						
	**GAF**		**Other clinical variables**				**Medication**

Shimodera et al. (73)	NA		n.s.				NA
Koike et al. (3)	Positive association in the FPC region in the chronic schizophrenia group		Positive association with PANSS positive or negative scores in the FEP group				n.s. (including no difference between UHR individuals with and without medication)
Takeshi et al. (72)	n.s., But positive association in the FPC during an idea fluency task		NA				NA
Azechi et al. (70)	NA		n.s.				n.s.
Quaresima et al. (69)	NA		NA				NA
Ikezawa et al. (71)	NA		n.s.				n.s.
Takizawa et al. (5)	Positive association in the FPC and right DLPFC regions		Negative correlation with age at measurement, positive correlation with PANSS positive score, and negative correlation with PANSS general psychopathology score in schizophrenia				n.s.
Ehlis et al. (74)	NA		NA				n.s.
Kubota et al. (75)	NA		NA				NA
Suto et al. (4)	NA		NA				NA
Watanabe and Kato (68)	NA		n.s.				n.s.

*Excluded previous gene association studies*.

*NA, not applicable; n.s., no significant correlation; FPC, the frontopolar prefrontal cortex; DLPFC, dorsolateral prefrontal cortex; VLPFC, ventrolateral prefrontal cortex; FDR, false discovery rate; GAF, the global assessment of functioning; PANSS, the positive and negative symptom scale; FEP, first-episode psychosis, UHR, ultra-high risk*.

*^a^ OMM-3000/16, Shimadzu Corporation; ETG-4000 and ETG-100, Hitachi Medical Corporation; NIRO-300 and NIRO-200, Hamamatsu Photonics Corporation; HEO-200, Omron Healthcare Corporation*.

*^b^ Exhibit case in the chronic schizophrenia group. Sixteen UHR and 2 FEP individuals were antipsychotics naïve and 8 UHR and 1 FEP individuals were drug naïve*.

*^c^ As Azechi et al. (70) used the same sample from Ikezawa et al. (71) as the First group, we describe for the Second group*.

*^d^ By Azechi et al. (70)*.

*^e^ Results from brain activity during the whole of the task period*.

*^f^ Results in the chronic schizophrenia group*.

*^g^ Results during the letter fluency tasks*.

The CFT has also been used in fNIRS studies, which often compare this task to the LFT. Kubota et al. used a two-channel fNIRS instrument to show for the first time that healthy controls had larger activity in the PFC during the LFT than in the CFT under similar task performances, whereas patients with schizophrenia had smaller activity during the LFT than during the CFT (75). Patients with schizophrenia had smaller activity than did healthy controls under similar task performances between the groups. This result is consistent with those of other studies (70, 71). Ehlis et al. used a multi-channel fNIRS instrument that covered the left frontotemporal region and found that healthy controls had larger and spatially wider activities during the LFT compared with the CFT (74), which was then replicated by measuring wider areas of the bilateral prefrontal/temporal cortices (76). Ehlis et al. also replicated the finding that patients with schizophrenia had significantly reduced activities compared with healthy controls during LFT, but not during the CFT.

### Application of fNIRS as a supplementary diagnosis tool for psychiatric disorders

As described above, previous studies using a block-design LFT have indicated that patients with schizophrenia have not only reduced activity, but also inappropriate activity timing, especially at the start of the task period and post-task period, compared with healthy controls (4, 5). Subsequently, the Joint Project for Psychiatric Application of Near-Infrared Spectroscopy (JPSY-NIRS) Group has applied to these results and improved in the applicable way for clinical settings. The integral value (the size of the fNIRS signal area during the task period) and centroid value (the centroid time of the fNIRS signal area throughout the task) were determined by using averaged brain signals estimated in the frontopolar cortex. This group showed preliminarily that 69% of patients with MDD and 69% of patients with schizophrenia, and 69% MDD patients and 81% BP patients were correctly differentiated under an algorithm using these two values (10, 11). A part of these results was approved as one of the Advanced Medical Technologies as an aid for the differential diagnosis of depressive symptoms in 2009, which was the first such approval in the field of psychiatry in Japan (10, 11). Several criticisms have arisen regarding the limited replication in various clinical settings and the lack of consensus for application to mental health (77, 78). However, paper published recently on JPSY-NIRS replicated previous results (12). Using this algorithm, fNIRS can differentiate patients with depressive symptoms between major depressive disorder and psychotic disorders (bipolar disorder and schizophrenia), with a high classification rate (74.6 and 85.5%, respectively).

### Other cognitive tasks and measurement settings

Three studies have explored the differences in brain activity during a random generation task (RNG) between patients with schizophrenia and healthy controls. Sinba et al. used a two-channel fNIRS instrument to describe for the first time that patients with schizophrenia had reduced brain activity in the PFC and worse task performances during the RNG compared with healthy controls, which represented different features during ruler-catching and sequential finger-to-thumb tasks (79). As healthy controls with better RNG task performances had greater brain activity, it remained unclear whether low brain activity was derived from worse task execution and/or functional impairment in schizophrenia. Hoshi et al. used time-resolved spectroscopy and two-channel fNIRS instruments to show that patients with schizophrenia, particularly those with a longer duration of illness, had reduced hemoglobin concentration during the resting state, and that this may cause altered activity during the RNG task (80). Koike et al. used a multi-channel fNIRS instrument to show that patients with schizophrenia had significantly reduced activity in the bilateral DLPFC and VLPFC regions, and that the activity in the right DLPFC region was associated with an earlier age at onset (81).

Okada et al. firstly showed the presence of altered brain activity in schizophrenia using a two-channel fNIRS instrument (62). Patients with schizophrenia had an aberrant task-related response pattern during a mirror drawing task, which was thought to be derived from a disrupted interhemispheric integration. Fallgatter et al. also showed the presence of altered frontal lateralization in schizophrenia during a continuous performance task using a two-channel fNIRS instrument (82). Folley et al. explored brain activity in patients with schizophrenia, individuals with schizotypal personality, and healthy controls during a divergent-thinking task using a two-channel fNIRS instrument, and showed that individuals with schizotypal personality had enhanced divergent-thinking ability and greater brain activity in the right PFC compared with patients with schizophrenia and healthy controls (83). Lee et al. showed alternations in brain activity using fMRI and 24-channel fNIRS (not simultaneously) during the same event-related spatial working-memory task: patients with schizophrenia recruited the bilateral PFC, whereas healthy controls recruited only the right PFC (28). Zhu et al. used a 48-channel fNIRS instrument to show that patients with first-episode schizophrenia had reduced brain activity over the PFC during the Tower of London task (84). Nishimura et al. used a 52-channel fNIRS instrument during a go-no-go task to show that healthy controls had a significant decrease in activity in the DLPFC during the no-go condition, whereas patients with schizophrenia exhibited no changes (67). Furthermore, the high excitement score observed in patients with schizophrenia was associated with brain activity in the FPC and right DLPFC. Taniguchi et al. used a 24-channel fNIRS instrument to show that patients with schizophrenia had reduced brain activity in the PFC compared with healthy controls during a kana Stroop task, with similar task performances, whereas both patients and controls showed lack of activity during a kanji Stroop task, with significantly worse performance observed in the schizophrenia group (85).

Other than cognitive tasks, Fujita et al. explored hemoglobin changes through an electroconvulsive therapy using a two-channel fNIRS instrument, and showed that patients with schizophrenia had asymmetric hemoglobin changes in the PFC compared with patients with depression (86).

### Genetic variation

Although schizophrenia is a syndrome, has been considered as a consolidation of several pathophysiological features, and has high genetic heritability, no crucial genetic risk factor has been found (87, 88). The results of genome-wide association studies that used a large sample size have suggested that schizophrenia risk genes are unable to be determined by specific gene variants but are thought to consist of common variants; furthermore, these risk genes had a substantial influence on environmental effects that occurred before the onset of schizophrenia. To clarify the impact of specific genes related to schizophrenia on the brain, imaging/genetics studies were performed to explore the relationship between brain structure and activity and genetic variants.

Five studies have explored the relationship between genetic variants and brain activity using fNIRS instruments. Takizawa et al. firstly reported that the val108/158met polymorphism of the catechol-*O*-methyltransferase (*COMT*) gene affected brain activity in the PFC only in schizophrenia patients (and not in healthy controls) (89). Schizophrenia patients with the Met variant (Val/Met and Met/Met) had significantly greater activation in the bilateral FPC and DLPFC during the LFT than did Val/Val carriers, implying that the inverted U curve shift of dopamine availability in schizophrenia might have an effect on the brain activity in the PFC (89). Regarding the Gln/Pro polymorphism of the sigma-1 receptor gene, Takizawa et al. reported that patients with the Gln/Gln genotype had significantly greater brain activity in the FPC and left DLPFC during the LFT than did Pro allele carriers; however, no significant differences were observed in healthy controls (90). However, Ohi et al. later used a two-channel fNIRS machine and a larger sample set to show that this genotype effect occurred in both the schizophrenia and control groups (91). Ohi et al. also reported that individuals with a longer cytosine/adenine/guanine (CAG) repeat in the spinocerebellar ataxia type 17 gene had reduced activity in the bilateral PFC during the TOH task in both the schizophrenia and control groups (92). Regarding the rs41279104 polymorphism of the nitric oxide synthase-I gene, Reif et al. showed that patients who were A allele carriers had significantly reduced activity in the right PFC during a VFT compared with those who had the GG genotype (93).

As recent methodological progress in gene analysis allows the exploration of whole genetic alterations between cases and controls using more than 10,000 samples, imaging/genetics studies should be performed using methods that enable the analysis of numerical data sets, such as bioinformatics and machine learning methods (94). Conversely, the investigation of the relationship between brain activity and relevant target genes (e.g., DLPFC function under dopamine regulation and *COMT* variants) may provide another imaging/genetics study strategy. The analysis of altered gene function in the brain among psychiatric illnesses may clarify the pathophysiology of specific psychiatric disorders and identify new treatment options.

### Effect of medication on brain activity

As previous clinical fNIRS studies have mostly explored patients in the chronic stage and receiving medication, the effect of medication on brain function was a limitation of these studies. Although an inconsistent effect of medication on fNIRS signal has been reported (68, 70, 71, 79), most previous fNIRS studies have reported an absence of association between brain activity and medication dose (3, 5, 12, 74, 81, 84–86) or different brain activity between individuals with ultra-high risk for psychosis with and without medication (3) (Table 1). However, all of those studies were cross-sectional, and a previous randomized and controlled trial showed that the administration of mirtazapine increased brain activity compared with trazodone and placebo in healthy volunteers (95). Controlled trials and/or longitudinal investigation to elucidate specific drug effects will be needed.

## Limitations of Previous Studies and Further Directions

The previous fNIRS studies of schizophrenia had several limitations; therefore, we propose further directions for future investigation (Table 3). First, as most previous studies have performed cross-sectional measurements in chronic and stable patients receiving medication, symptomatic, and functional changes were not fully explored. As fNIRS is able to perform measurements relatively easily in unstable patients, such as those with acute or recurrent conditions, longitudinal studies aimed at investigating changes in clinical symptoms and social function will be needed (3, 96). Although previous studies have revealed a negligible medication effect on fNIRS signals, investigations of drug-naïve patients or of those receiving controlling treatment (e.g., specific drugs, electroconvulsive therapy, and neurofeedback) will be needed to allow further clinical applications of fNIRS (60, 86, 97). The use of the easy portability of fNIRS machines may allow measurements in earlier clinical stages in cohort settings, such as adolescents with psychotic-like experiences, which may reveal the alterations in brain development in the PFC (98).

**Table 3:** **Limitation of previous fNIRS studies and further implication**.

Limitation of previous studies	Further implications
Cross-sectional measurement for chronic and stable patients with medication	Measure in different clinical stages (e.g., UHR, FEP, recurrent phase)
	Measure longitudinally to explore the relationship between clinical changes and fNIRS signals
	Measure in drug-naïve patients to explore drug effect
	Measure in a specific treatment response (e.g., medication, electroconvulsive therapy, neurofeedback)
	Measure in cohort setting (e.g., psychotic-like experiences)
One-sided task procedure (block-designed VFTs)	Use of other cognitive tasks
	Adopt event-related design
	Adopt more naturalistic task (e.g., driving, conversation)
Small sample size in single institute	Make consortium with the same instrument and measurement procedure (e.g., task paradigm, probe setting)
	Adopt more sophisticated analytical methods (e.g., measurement tools, bioinformatics, machine learning)
	Standardize manuscript description
	Construct fNIRS community

*UHR, ultra-high risk; FEP, first-episode psychosis*.

Second, half of the previous studies of schizophrenia adopted block-design VFTs for cognitive activation. Although one of the major disadvantages of fNIRS is the inability to measure brain activity in deep brain tissues, and the block-design VFT is appropriate for elucidating brain activity over the PFC, other cognitive tasks, and event-related design will be helpful to explore brain pathology in schizophrenia and to compare signal differences between fNIRS and other neuroimaging tools, such as fMRI and PET. Conversely, the exploration of brain activity in a more naturalistic position and during natural activities, such as driving and conversation, is suitable for future investigations using the advantages of fNIRS (99, 100).

Third, as fNIRS allows easy and repetitive measurements, a study including a large number of samples has been conducted (12). To analyze such a large data set, the same measurement procedure regarding task paradigm and probe setting and more sophisticated analytical methods, such as measurement tools (e.g., NIRS-SPM) (101), bioinformatics methods, and machine learning (94), will be needed. Standardized manuscript description and a database system will be needed for further comparisons and meta-analyses (102). The construction of an fNIRS community is expected to provide this type of background in NIRS research (18).

## Conclusion

Functional near-infrared spectroscopy has been progressing rapidly in the field of psychiatry, as it provides several advantages, such as small size, portability, silent functioning, and the achievement of easy and non-invasive measurements. A part of these results was approved in 2009 as one of the Advanced Medical Technologies as an aid for the differential diagnosis of depressive symptoms (10–12), which was the first such approval in the field of psychiatry in Japan. Future investigations aimed at exploring fNIRS differences in various clinical stages, longitudinal changes, drug effects, and variations during different task paradigms will be needed to develop more accurate biomarkers that can be used to aid differential diagnosis, the comprehension of the present condition, the prediction of outcome, and the decision regarding treatment options in schizophrenia. Future fNIRS research environments will require standardized measurement procedures, probe settings, analytical methods and tools, manuscript description, and database systems in a n fNIRS community.

## Conflict of Interest Statement

The authors declare that the research was conducted in the absence of any commercial or financial relationships that could be construed as a potential conflict of interest.
